# An App-Based Intervention With Behavioral Support to Promote Brisk Walking in People Diagnosed With Breast, Prostate, or Colorectal Cancer (APPROACH): Process Evaluation Study

**DOI:** 10.2196/64747

**Published:** 2025-02-10

**Authors:** Fiona Kennedy, Susan Smith, Rebecca J Beeken, Caroline Buck, Sarah Williams, Charlene Martin, Phillippa Lally, Abi Fisher

**Affiliations:** 1 Leeds Institute of Health Sciences University of Leeds Leeds United Kingdom; 2 Department of Behavioural Science and Health University College London London United Kingdom; 3 Division of Clinical Medicine School of Medicine and Population Health University of Sheffield Sheffield United Kingdom; 4 School of Psychology University of Surrey Guildford United Kingdom

**Keywords:** cancer, physical activity, process evaluation, randomized controlled trial, intervention, app, habit

## Abstract

**Background:**

The APPROACH pilot study explored the feasibility and acceptability of an app (NHS Active 10) with brief, habit-based, behavioral support calls and print materials intended to increase brisk walking in people diagnosed with cancer.

**Objective:**

Following UK Medical Research Council guidelines, this study assessed the implementation of the intervention, examined the mechanisms of impact, and identified contextual factors influencing engagement.

**Methods:**

Adults (aged ≥18 y) with breast, prostate, or colorectal cancer who reported not meeting the UK guidelines for moderate-to-vigorous physical activity (≥150 min/wk) were recruited from a single hospital site in Yorkshire, United Kingdom. They were randomly assigned to the intervention or control (usual care) arm and assessed via quantitative surveys at baseline (time point 0 [T0]) and 3-month follow-up (time point 1 [T1]) and qualitative exit interviews (36/44, 82%) at T1. The process evaluation included intervention participants only (n=44). Implementation was assessed using data from the T1 questionnaire exploring the use of the intervention components. The perceived usefulness of the app, leaflet, and behavioral support call was rated from 0 to 5. Behavioral support calls were recorded, and the fidelity of delivery of 25 planned behavior change techniques was rated from 0 to 5 using an adapted Dreyfus scale. Mechanisms of impact were identified by examining T0 and T1 scores on the Self-Reported Behavioural Automaticity Index and feedback on the leaflet, app, call, and planner in the T1 questionnaire and qualitative interviews. Contextual factors influencing engagement were identified through qualitative interviews.

**Results:**

The implementation of the intervention was successful: 98% (43/44) of the participants received a behavioral support call, 78% (32/41) reported reading the leaflet, 95% (39/41) reported downloading the app, and 83% (34/41) reported using the planners. The mean perceived usefulness of the app was 4.3 (SD 0.8) in participants still using the app at T1 (n=33). Participants rated the leaflet (mean 3.9, SD 0.6) and the behavioral support call (mean 4.1, SD 1) as useful. The intended behavior change techniques in the behavioral support calls were proficiently delivered (overall mean 4.2, SD 1.2). Mechanisms of impact included habit formation, behavioral monitoring, and support and reassurance from the intervention facilitator. Contextual factors impacting engagement included barriers, such as the impact of cancer and its treatment, and facilitators, such as social support.

**Conclusions:**

The APPROACH intervention was successfully implemented and shows promise for increasing brisk walking, potentially through promoting habit formation and enabling self-monitoring. Contextual factors will be important to consider when interpreting outcomes in the larger APPROACH randomized controlled trial.

**International Registered Report Identifier (IRRID):**

RR2-10.1186/s40814-022-01028-w

## Introduction

### Background

The number of people being diagnosed with cancer is continuing to increase in the United Kingdom, with an estimated 4 million adults living with and beyond cancer (LWBC) by 2030 [1]. Many people LWBC experience challenges related to cancer and its treatment, including increased fatigue, pain, psychological distress, and reduced physical capacity [2-5]. These challenges can significantly impact their quality of life and well-being [6]. Therefore, the importance of improving outcomes for those LWBC is vital [7]. A large body of trial data demonstrates that physical activity (PA) improves many outcomes after a cancer diagnosis, including reduced fatigue, pain, anxiety, depression, and sleep problems and an overall improvement in quality of life [8-11]. Observational data suggest that PA is also associated with improvements in survival [12-14]. In light of this ever-growing evidence base, the World Cancer Research Fund recommends that people LWBC follow the guidelines for healthy populations in achieving at least 150 minutes of moderate-to-vigorous PA (MVPA) per week and recommend limiting the amount of time spent sedentary [15]. Despite this, many people LWBC are physically inactive, with Macmillan Cancer Support (United Kingdom) estimating this to be as high as 80% of those LWBC not meeting recommended PA levels [16]. This is supported by the systematic review of 41 studies conducted by Wong et al [17] that indicated that only around a third of people LWBC were meeting PA guidelines, although this ranged from 16% to 88% across studies. Brisk walking is a form of MVPA that may be more appropriate for people LWBC due to its accessibility and achievability. In a systematic scoping review of 98 studies in people LWBC, walking was cited as the preferred type of PA across all cancer types and treatment stages [18].

### The APPROACH Intervention

APPROACH is an app-based, multicomponent intervention informed by extensive development work with individuals with breast, prostate, or colorectal cancer and cancer nurse specialists [19,20]. It focuses on promoting and monitoring brisk walking using a publicly available mobile phone app, alongside brief behavioral support in the form of a specially designed leaflet, walking planner cards, and 2 phone or video calls with a trained researcher (CB) [21]. In line with the Medical Research Council framework for intervention development and evaluation, the pilot trial explored the feasibility and acceptability of conducting a complex PA intervention trial with people LWBC. A total of 90 people diagnosed with breast, prostate, or colorectal cancer were recruited for the pilot randomized controlled trial (RCT), with 49% (n=44) randomly assigned to the intervention group and 51% (n=46) to the control group. APPROACH pilot results demonstrated a high retention rate (97%) and high assessment completion rates (>86%), indicating that the trial procedures were feasible and acceptable to be carried out as intended in a confirmatory, phase-3, larger trial [22]. In addition, results showed that the intervention was delivered successfully with 98% receiving at least 1 behavioral support call and 95% of participants downloading the app [22].

### Conducting a Process Evaluation

The importance of conducting process evaluations within RCTs has been emphasized to explore the way in which any complex intervention is implemented [23,24]. This can help uncover why interventions are successful or unsuccessful, determine why they may have unexpected consequences, as well as explore how an intervention that is effective can be optimized [23]. Moore et al [25] provided specific guidance for carrying out process evaluations, which highlighted the importance of exploring the implementation (eg, the fidelity of intervention calls), the mechanisms of impact (eg, views on the different components of the intervention), and the contextual factors influencing use and outcomes (eg, barriers or facilitators to engagement) [25].

Therefore, this paper extends the published APPROACH feasibility results [22] with the following aims: (1) to evaluate the implementation of the APPROACH intervention and the fidelity of the delivery of intended behavior change techniques (BCTs), (2) to identify the potential mechanisms of impact that underlie behavior changes attributed to the APPROACH intervention, and (3) to better understand how contextual factors influence engagement with the APPROACH intervention.

## Methods

### Design

This was an embedded design, mixed methods study where qualitative and quantitative data were collected simultaneously with equal priority [26]. Data were collected as part of the APPROACH pilot RCT [21,22]. The pilot RCT compared an app-based, brisk walking intervention delivered alongside usual care with a control arm (usual care alone) in people diagnosed with breast, prostate, or colorectal cancer at a single hospital site. The trial was registered on the International Standard Randomized Controlled Trial Number registry on April 16, 2021 (ISRCTN 18063498). The primary outcome for the pilot and future RCT is weekly minutes spent brisk walking (a cadence of 100 steps per min or more [27) measured by an activPAL accelerometer (PAL Technologies Ltd). Following baseline assessments, participants were individually randomly assigned (1:1 allocation) using minimization to either the control or intervention arm, stratified by cancer type (breast, prostate, or colorectal) and disease status (metastatic vs not).

### Participants

The pilot RCT included 90 participants: 49% (n=44) in the intervention arm and 51% (n=46) in the control arm. All participants had a confirmed diagnosis of breast, prostate, or colorectal cancer (localized or metastatic). At the point of screening, localized participants had to be within 6 months of completion of radical treatment. This criterion was not applied to participants with metastatic disease. All participants required a clinician’s sign-off that their life expectancy was >6 months. All participants self-reported achieving <150 minutes of MVPA weekly. Full inclusion and exclusion criteria have been published previously [21].

### Procedure and Description of Intervention

The recruitment procedure and trial data collection have previously been described [21,22]. Participants completed assessments at baseline (time point 0 [T0]) and 3 months (time point 1 [T1]; operationalized as 12-16 wk after randomization). The intervention included an endorsement letter from a member of the participant’s clinical team, alongside the provision of a leaflet with information about the importance of PA after cancer and a recommendation and instructions on how to download the freely available NHS Active 10 app. The NHS Active 10 app promotes brisk walking in bouts of 10 minutes, called “Active 10s.” This was augmented with 2 behavioral support phone or video calls with the intervention facilitator (CB). The app was chosen after previous qualitative work with people LWBC, and clinicians identified key features that were important to be offered within the app and highlighted the importance of the app being supported by a professional organization, such as the NHS [19,20]. These behavioral support calls were underpinned by habit theory [28] and BCTs shown to be effective in promoting PA [29-31] and involved supporting participants in downloading and using the app and discussions around setting PA goals. The intervention facilitator was trained in the principles of behavior change theories and in the application of BCTs [32], with a thorough understanding of habit theory [33]. This training, alongside previous experience in delivering health behavior change interventions, allowed them to conduct conversations with patients closely mirroring those a health care professional might have with a patient in a routine care situation. The intervention calls took place via Zoom (Zoom Video Communications) [34] or telephone and were recorded by the intervention facilitator with the participants’ permission. The first call took place approximately 1 week after randomization, and the second call took place approximately 4 weeks after the first call to check in with the participant about their goals and recap any information required. Participants were asked during the first intervention call if they had downloaded the NHS Active 10 app, and the intervention facilitator noted this in their records. Participants were also given 12 copies of a walking planner card that was designed to enable them to plan how many “Active 10s” they were aiming for and how they were going to achieve these, including where and when they would complete them.

### Implementation of the APPROACH Intervention

#### Delivery of the Intervention

The implementation of the intervention was explored by looking at whether each component was delivered as intended and the participants’ use of each intervention component. Between 12 and 16 weeks after randomization participants completed the T1 questionnaire. Participants were asked about the intervention, including the following: whether they downloaded the app (yes or no), their self-reported app use if still using the app (less than monthly, monthly, fortnightly, weekly, 3-4 times a week, almost every day, or every day), how long they used the app for if they had stopped using it (never, once, less than monthly, fortnightly, weekly, 3-4 times per week, almost every day, or every day), perceived accuracy of the app in recording their time spent walking (5-point scale from not accurate to very accurate), whether they read the leaflet (all, some, or did not read), used the walking planner cards (yes or no), and received either behavioral support call (yes or no).

#### The Usefulness of Intervention Components

Participants rated the usefulness of the call for going through the leaflet information, downloading the app, and thinking about ways to use the app to increase their brisk walking (5-point scale from not at all useful to extremely useful). Using the same scale, they rated the usefulness of the app and sections of the leaflet for supporting their walking.

#### Delivery Fidelity of Behavioral Support Calls

The recorded intervention calls were coded by 1 researcher (SW) to assess delivery fidelity. All calls were listened to and coded according to a 25-item checklist of BCTs [31] as presented in the study’s protocol paper [21]. Each item represented a BCT paired with the intended delivery technique (Multimedia Appendix 1). If a participant received 2 calls, these were combined when coding delivery of the BCT. A 5-point rating scale was applied to the fidelity checklist using an adaptation of the Dreyfus scale [35,36] ranging from low fidelity (0), indicating that the facilitator did not mention the intended BCT at all, to expert (5), indicating that the facilitator delivered the BCT to an exceptional standard (Multimedia Appendix 2). A value of ≥3 represented competent delivery of an individual BCT, thus presenting successful delivery. A second researcher (SS) coded a subset of interviews (n=5). It was agreed that if there was a discrepancy of over 20% in the coding, then the transcript would be discussed among the researchers. This occurred for 1 transcript that was double coded. This iterative process enabled SW to incorporate any learnings from the discussion into the coding of all transcripts and allowed a more consistent coding of the data.

### Mechanisms of Impact and Contextual Factors Influencing Engagement

#### T0 and T1 Questionnaires

Habit strength for walking (“going for a walk” and “walking briskly”) was assessed using the Self-Report Behavioural Automaticity Index (SRBAI) [37] in the T0 and T1 questionnaires. Participants responded on a 7-point scale ranging from disagree to agree for 4 statements on their perceived automaticity of performing the behavior. An average score across items was calculated, representing the level of automaticity for the behavior being measured. Higher average scores indicate stronger habit or greater automaticity [37]. The SRBAI is presented in Multimedia Appendix 3. Mechanisms of impact and contextual factors impacting engagement were also identified by examining responses to the T1 questionnaire about the delivery of intervention components and their perceived usefulness.

#### Qualitative Interviews

Participants were asked in the initial study consent form if they agreed to be invited to participate in a semistructured interview at the end of the study. After the completion of all other data collection at T1, all participants who agreed were invited to be interviewed. Two members of the research team (SS and FK) carried out the interviews. SS and FK were involved in organizing assessments with participants throughout the pilot RCT. The interviews followed a topic guide exploring trial procedures, and participants were asked to give feedback on the intervention components (Multimedia Appendix 4). Interviews took place over the phone and, with the participants’ permission, were audio recorded and transcribed verbatim. Contextual factors were explored in the interviews and were described in terms of barriers and facilitators of engaging with the intervention.

### Data Analysis

#### Implementation of the APPROACH Intervention

The T1 questionnaire responses on intervention components were explored descriptively by calculating percentage frequencies and, where relevant, measures of central tendency. Mean scores were calculated for the delivery of each BCT in the intervention calls as well as an overall mean fidelity score for each call.

#### Mechanisms of Impact and Contextual Factors Influencing Engagement

The data from the questionnaires and interviews were pooled during interpretation to investigate the mechanisms of impact and contextual factors of intervention engagement. Qualitative and quantitative data were analyzed separately. The SRBAI results from the T0 and T1 questionnaires were explored descriptively using medians and IQRs due to the skewness of the data. The T1 questionnaire responses on intervention components were also used to identify mechanisms of impact.

Three authors (SW, FK, and SS) analyzed the data from the qualitative interviews using reflexive thematic analysis [38,39]. Inductive coding was undertaken, with these codes then used to develop themes, and early and final themes were discussed throughout the coding process among multiple authors (FK, SS, and PL). While initial coding was inductive and focused on identifying commonalities across the transcripts, final theme development was also organized by focusing on the outlined process evaluation aims on exploring the delivery of the intervention, the mediating processes (mechanisms of impact), and the barriers and facilitators to engagement (contextual factors). All interview transcripts were managed in NVivo (version 12; Lumivero) to facilitate analysis and data management.

Data were integrated using a complementarity approach where the interpretation of quantitative and qualitative results together allowed a more holistic interpretation of the findings [40].

### Ethical Considerations

This pilot study was approved by the Yorkshire & The Humber-South Yorkshire Research Ethics Committee (21/YH/0029, Health Research Authority and the local hospital. All participants gave informed consent and the data reported are anonymized.

## Results

### Overview

Table 1 presents the sample characteristics of the 44 participants in the intervention arm. Most (42/44, 95%) of the participants were of White ethnicity, comprising an equal number of male participants (22/44, 50%) and female participants (22/44, 50%), with a mean age of 63 (SD 11; range 40-85) years. Participants had received a diagnosis of breast cancer (18/44, 41%), prostate cancer (18/44, 41%), or colorectal (8/44, 18%) cancer. The CONSORT (Consolidated Standards of Reporting Trials) diagram and the flow of participants through the study have previously been reported [22]. After eligibility screening and assessment of interest in taking part, the study information sheet was sent to 148 patients, and 63% (n=93) consented to participate, with 61% (n=90) being randomized.

**Table 1:** Characteristics of the APPROACH intervention group (N=44).

Characteristics		Participants, n (%)
**Sex**		
	Male	22 (50)
	Female	22 (50)
**Age range (y)**		
	40-50	7 (16)
	51-60	10 (23)
	61-70	14 (32)
	71-80	12 (27)
	>81	1 (2)
**Ethnic group**		
	Asian	1 (2)
	Black	0 (0)
	Mixed	0 (0)
	Other	1 (2)
	White	42 (95)
**Cancer type**		
	Breast	18 (41)
	Prostate	18 (41)
	Colorectal	8 (18)
**Localized or metastatic**		
	Localized	41 (93)
	Metastatic	3 (7)
**Relationship status**		
	Married or in a relationship	37 (84)
	Single, divorced or separated	3 (7)
	Widowed	4 (9)
**Employment**		
	Full time	8 (18)
	Part time	9 (20)
	Unemployed	2 (5)
	Retired	22 (50)
	Unable or too ill to work	3 (7)
**Living arrangements**		
	Alone	5 (11)
	With partner	25 (57)
	With family	14 (32)
**Index of Multiple Deprivation quintile**		
	1 (most deprived)	8 (18)
	2	6 (14)
	3	9 (20)
	4	16 (36)
	5 (least deprived)	5 (11)

### Implementation of the APPROACH Intervention

#### Overview

In total, 2 (5%) of the 44 participants withdrew from the intervention group for reasons unrelated to the intervention (frustration with the accelerometer and increased caring responsibilities). Most (41/42, 98%) participants who remained in the study answered the section of the T1 questionnaire on intervention feedback. Moreover, 1 (2%) of the 42 participants did not complete this section on intervention feedback.

#### Delivery of the Intervention

##### Leaflet

In the T1 questionnaire, 78% (32/41) of the intervention participants reported reading the entire intervention leaflet, while 10% (4/41) reported reading some of it, and 12% (5/41) reported not reading it at all. Of those who did not read it at all, 80% (4/5) stated that they did not remember receiving the leaflet, and 20% (1/5) stated that it was not relevant to them.

##### NHS Active 10 App

At the time of the first behavioral support phone call, the intervention facilitator recorded that 95% (42/44) participants had downloaded the NHS Active 10 app, with 93% (39/42) independently downloading it before the first intervention call and 7% (3/42) downloading it during the call. However, 2% (1/43) of the participants left call 1 not having downloaded it. In the T1 questionnaire, 95% (39/41) of the intervention participants self-reported successfully downloading the app.

In total, 5% (2/41) of the participants were not asked about their use of the app as they reported not downloading the app earlier in the questionnaire. Most (33/39, 85%) participants reported still using the app. Of these, 82% (27/33) reported using it “almost every day or every day,” and 18% (6/33) reported that they used it “3-4 times per week.” A few (5/41, 12%) participants reported using the app during the study but were no longer using it. When asked how long they had used the app, the participants reported using it for “1 week,” “2 weeks,” “1 month,” “2 months,” and “3 months.” In addition, 2% (1/41) of the participants reported not using the app at all despite downloading it.

##### Planner Cards

In the T1 questionnaire, 83% (34/41) of the participants reported using the walking planner cards, whereas 17% (7/41) did not, including 1 participant who said they did not receive any cards. Other nonuse was mainly explained in terms of not finding it helpful/not needing to plan (5/41, 12%) or having a more physical job (1/41, 2%). Of those who used the planners, 65% (22/34) reported using the planners for the full 3 months, whereas others reported using them for 2 weeks (4/34, 12%), 1 month (4/34, 12%), or 2 months (4/34, 12%).

#### The Usefulness of Intervention Components

##### Overview

The perceived usefulness of the intervention components is presented in Table 2.

**Table 2:** Perceived usefulness of the APPROACH intervention components (n=41).

Intervention components^a^			Values, mean (SD)		Respondents, n (%)
Behavioral support call			4.1 (1.0)^b^		40^c^ (98)
**Leaflet sections**					
	Physical activity and cancer	3.8 (0.9)^b^		36^d^ (88)	
	Walking	4.0 (0.8)^b^		36^d^ (88)	
	Information about Active 10	3.9 (0.8)^b^		36^d^ (88)	
	Instructions on how to download Active 10	4.0 (0.8)^b^		36^d^ (88)	
	Walking habits	4.1 (0.7)^b^		36^d^ (88)	
	Walking websites	3.8 (1.5)^b^		36^d^ (88)	
Mean usefulness of leaflet sections			3.9 (0.6)^b^		36^d^ (88)
App usefulness in participants still using the app			4.3 (0.8)^b^		33^e^ (80)
App usefulness in participants who had stopped using the app			2.6 (0.9)^b^		5^e^ (12)
App accuracy in participants still using the app			3.9 (1.2)^f^		33^e^ (80)
App accuracy in participants who had stopped using the app			2.2 (0.8)^f^		5^e^ (12)

^a^The perceived usefulness of the walking planner card was not explored in the time point 1 questionnaire (n=41).

^b^A 5-point scale from not at all useful to extremely useful.

^c^One person reported not receiving a behavioral support call and was not shown this question.

^d^Five people reported that they had not read the leaflet and were not shown these questions.

^e^One participant reported downloading but never using the app to track their walking. Two participants self-reported not downloading the app earlier in the questionnaire. These participants were not shown this question.

^f^A 5-point scale from not accurate to very accurate.

##### Delivery Fidelity of Behavioral Support Calls

Most (43/44, 98%) of the participants received the first behavioral support call. The mean time from randomization to the first intervention call was 11.6 (SD 9.8; range 5-57) days, and the mean time from randomization to the second intervention call was 39.2 (SD 9.0; range 33-78) days. In total, 31 (72%) of the 43 calls were conducted on Zoom and 12 (28%) via telephone. Most (40/41, 91%) of the participants received the second call. Some (22/40, 55%) of these calls were conducted on Zoom and 45% (18/40) via telephone. In total, 81 intervention calls from 42 participants were included in the analysis (n=42, 52% first calls and n=39, 48% second calls). One intervention participant did not receive any calls, and another participant was removed from the analysis due to a recording issue with the first call, so neither of their calls was included in the fidelity results. The overall mean delivery fidelity score across all BCTs and all participants was 4.2 (SD 1.2), which demonstrates overall proficient delivery. Some (18/25, 72%) of the BCTs had a rating of >4, 16% (4/25) had a rating of 3 to 4, and 12% (3/25) had a rating <3. The BCT called provide information on health consequences had the highest fidelity (4.98). This was followed by action planning (4.90) and habit formation (4.88). The BCT called framing/reframing had the lowest fidelity (1.31). Nonspecific reward (2.71) and nonspecific incentive also displayed low fidelity. The delivery fidelity of each BCT that was intended to be delivered during the calls is presented in Multimedia Appendix 1.

### Mechanisms of Impact and Contextual Factors Influencing Engagement

**Figure 1:**
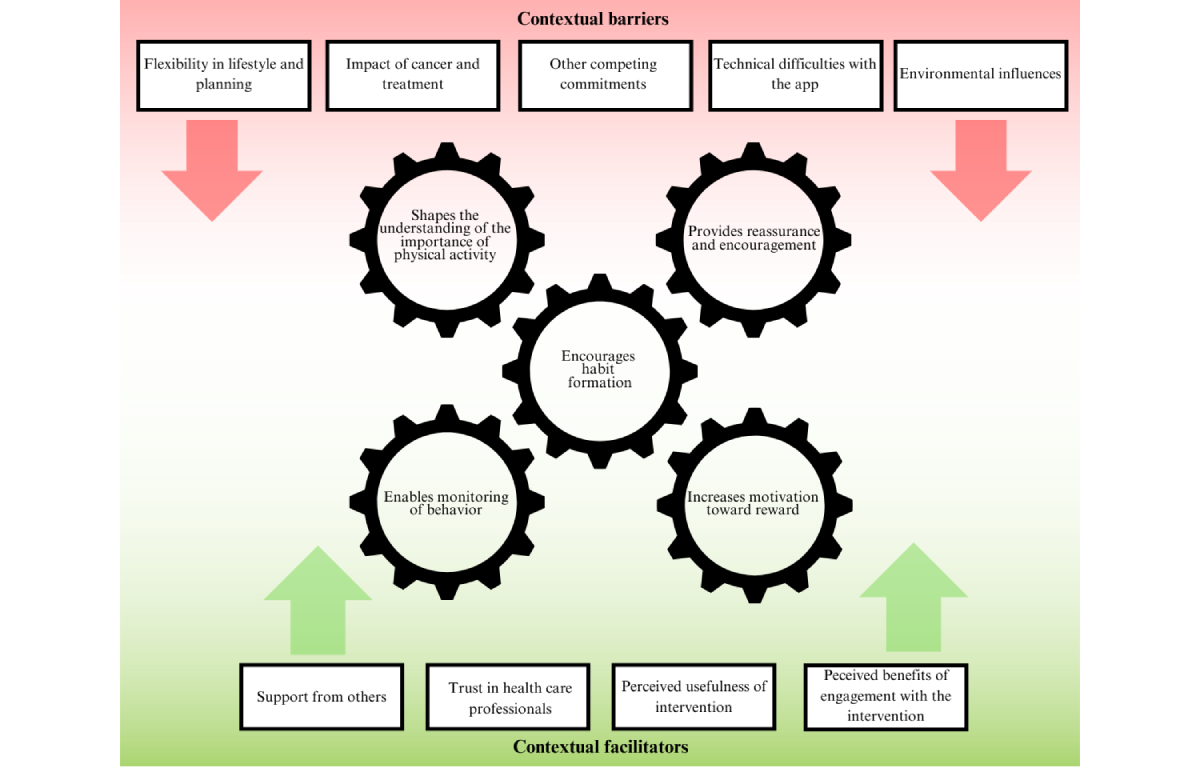
Theme diagram presenting the identified mechanisms of impact and the contextual barriers and facilitators that affect these mechanisms.

Figure 1 presents the theme diagram showing the identified mechanisms of impact and the contextual barriers and facilitators that affect these mechanisms and intervention engagement.

#### T0 and T1 Questionnaires

All (44/44, 100%) intervention participants completed the SRBAI at baseline. All (42/42, 100%) participants who remained in the study completed the SRBAI at T1. As mentioned earlier, most (41/42, 98%) of the participants who remained in the study answered the section of the T1 questionnaire on intervention feedback.

#### Qualitative Interviews

Of the 42 participants who remained in the study, 86% (n=36) took part in the qualitative interviews. A few (n=3, 7%) participants did not give a reason for declining to participate, and 2 (5%) participants consented to the interview but then did not respond to the interview invitation. Moreover, 1 (2%) participant did not feel up to taking part in the interview due to illness-related side effects.

#### Mechanisms of Impact

Identified mechanisms of impact are outlined in the subsequent sections with exemplar quotes indicating the participants’ self-identified sex (male or female) and age.

##### Shapes Understanding of PA and Its Importance

Many participants reported gaining information about brisk walking and its benefits as well as information on how to use the app through the intervention call and the leaflet:

...the lady basically went through everything. That was probably the most helpful thing. How to use things and everything.

Female participant; aged 42 y

The delivery of comprehensive and meaningful information enhanced participants’ understanding of the target behavior and provided them with a clear purpose for implementing it:

She went thoroughly through the app with me...Then when she started to explain it, I thought yes, that makes sense.

Male participant; aged 60 y

##### Enables Monitoring of Behavior

Participants reported that having 2 intervention calls was motivating as it helped them to reflect on their progress between the calls:

...the follow up call halfway through was touching base and seeing how I was getting on which obviously encouraged me to do it.

Male participant; aged 73 y

The feedback on behavior received via the app showing daily, weekly, and monthly minutes of brisk walking was considered effective. Participants reported that they found the tracking feature motivated them to continue their walking efforts:

And actually, when I realised I wasn’t doing enough, when I felt able to, I extend my walk to get the thirty minutes.

Female participant; aged 60 y

Several participants reported using the planner cards to record their walks afterward rather than planning with them upfront. However, this sense of accountability through recording their activity engaged them to keep walking:

I wrote down what was on my app, every day, how many minutes walking I did everyday.

Female participant; aged 62 y

##### Increases Motivation Toward Rewards

Many participants reported that the app was the primary intervention component that kept them most motivated and engaged, particularly through the trophies or cups awarded (for every 10 min of brisk walking):

I did enjoy getting them cups every day, I thought that were great.

Female participant; aged 61 y

Participants often reported walking a few more minutes to achieve the next reward or cup on the app, some even referring to being obsessed or addicted to achieving their goals:

...if I get to say 28 minutes, I’ll just do the extra two to make it thirty.

Female participant; aged 49 y

30 has been the minimum goal I’ve gone for. So even if it’s not been a nice day or if I’m tired...I still go out...it’s addictive.

Male participant; aged 75 y

The achievability of these rewards influenced engagement, with several participants reporting exceeding their targets and wishing that more rewards were available:

That’s another downside, you can’t set your goal to any more than three.

Female participant; aged 61 y

Participants also reported a sense of satisfaction when being able to tick off completing their walks in their planners:

...you can see something, you’re achieving something.

Female participant; aged 47 y

##### Encourages Habit Formation

Many participants reported feeling that they had formed habits throughout the intervention period, and this enabled them to establish and maintain their walking and brisk walking habits:

It’s part of it now, it’s part of your day, it’s part of your walk so its not I’m going I’ve got to do this, I’ve got to do that...It’s just a normal day for us going for a walk. And you get back and you think, mmm, I didn’t realise I was doing that quick.

Female participant; aged 65 y

This is also supported by the SRBAI results for “walking,” where total SRBAI scores in the intervention group increased from baseline (mean 4.1, SD 1.6) to T1 (mean 4.7, SD 1.9). Similarly, total SRBAI scores for “brisk walking” in the intervention group increased from baseline (mean 4.0, SD 1.7) to T1 (mean 5.1, SD 1.8).

##### Providing Reassurance and Encouragement

Many participants recalled how helpful and friendly the facilitator was in the intervention calls:

...they were lovely, caring and friendly.

Female participant; aged 61 y

This positive rapport helped participants adhere to their brisk walking, and they recalled feeling encouraged and supported throughout the intervention period:

...it’s useful because it makes you feel as if you’re not forgotten.

Male participant; aged 73 y

#### Contextual Factors

The contextual factors influencing engagement are summarized according to barriers and facilitators with exemplar quotes indicating the participants’ self-identified sex (male or female) and age.

##### Barriers

###### Flexibility in Lifestyle and Planning

Data from the qualitative interviews and the T1 questionnaire indicated that participants felt the planners, in their intended use (to plan walks each week), were not flexible enough and did not fit their lifestyles:

...it didn’t work for me at all because every day is different.

Female participant; aged 42 y

Related to this, some participants suggested making the planners daily, rather than weekly, which would allow more nuanced plans to be made:

...it wasn’t going to be at the same time every day so it just needed breaking down into a daily thing.

Female participant; aged 57 y

###### The Impact of Cancer and Its Treatment

Several participants reported in the interviews and the T1 questionnaire that their cancer diagnosis and treatment sometimes made it difficult to engage with the intervention:

...this was just after my treatment and I was tired a bit.

Female participant; aged 67 y

I couldn’t plan, because I was in and out of different appointment times.

Female participant; aged 57 y

This was also seen when participants were asked about the appropriateness of the app for people LWBC, with several participants reflecting on how differing experiences may help or hinder engagement:

I had some days where I felt like I didn’t want to see daylight, not talk to anybody...I think on those bad days I wouldn’t have wanted to be bothered with it.

Female participant; aged 67 y

Many participants discussed that the appropriateness of the timing of the intervention would be dependent on where the patient was in their cancer care. Although many participants noted that taking part in the intervention during treatment would have been too difficult for them due to the side effects, several also suggested that using the intervention during treatment was suitable as it gave them something else to focus on:

No I wouldn’t have been able to do it when I was having chemo, I could barely even walk round the garden.

Female participant; aged 58 y

This gave me something else to think about you know something on a daily basis which took me mind off you know that three-week cycle as much as it could.

Male participant; aged 65 y

###### Other Competing Commitments

Several participants reported not having time to walk due to factors, such as having caring responsibilities, working hours, and appointments:

...well the kids can’t walk with me they have only got little legs, who is going to watch them?

Female participant; aged 41 y

###### Technical Difficulties With the App

In the interviews, several participants reported experiencing difficulties with the recording of walks on the app if there was a lack of signal or due to the phone’s positioning. For example, sometimes participants found the recording differed depending on what pocket their phone was in:

...if I had it in my trouser pocket by my leg it worked all fine, ok, no problem at all, if I had it in my shirt pocket it didn’t register anything.

Male participant; aged 73 y

This is supported by the T1 questionnaire results indicating that perceived app accuracy was an issue that likely influenced use and engagement with the app over time:

Didn’t find the app as accurate as it could be and doubted its recordings on occasions.

Female participant; aged 41 y

###### Environmental Influences

Participants described how the weather played an influential role in their ability and motivation to go on their walks:

What it’s going to be like when it starts raining and it’s really awful weather I don’t know.

Female participant; aged 40 y

As well as hindering their ability to go out on a walk, some participants also described feeling guilty if they did not go out and walk due to the weather:

There were some days where it was absolutely throwing it down outside…and I thought oh no and I really felt guilty that I’d not actually done it.

Female participant; aged 61 y

##### Facilitators

###### Support From Others

Many participants talked about telling people of their involvement in the trial and their efforts to increase their walking, describing a sense of accountability from sharing the experience with others:

...so now it’s a case of, “Have you got your minutes in yet dad?” and it, everyone’s sort of like joining in with it.

Male participant; aged 75 y

In addition, having someone to go on a walk with and even having family members and friends also use the app was described as encouraging and gave participants a sense of comradery in changing their behavior:

It made us all as a family go for a walk. It not just helped me with that. It helped all of us.

Female participant; aged 42 y

Personal contact from the study team also facilitated engagement, with several participants reporting that they felt supported and that they had the opportunity to get in touch if needed:

...it were good to know that somebody you know, I’d phone and they’d take an interest.

Female participant; aged 61 y

###### Trust in Health Care Professionals

Several participants noted that endorsement from their health care professional facilitated their engagement with the intervention and willingness to change their behavior, as their medical team is seen as a credible source of information:

So the fact it had come from the doctor made me want to do it even more.

Female participant; aged 42 y

###### Perceived Usefulness of the Intervention

The perceived usefulness of the intervention components appeared to influence engagement in the target behavior, with participants reporting that some components (eg, app) were more helpful than others (eg, planners).

Overall, the behavioral support call was rated as useful (mean 4.1, SD 1; Table 2). In the qualitative interviews, the participants reported finding the calls useful as a source of information and motivation as well as helping them to regain their focus:

You know, if you’ve got an issue you can talk to somebody about it. But also it keeps you motivated.

Female participant; aged 56 y

The leaflet was generally rated as useful (mean 3.9, SD 0.6), particularly the sections on “walking,” “downloading the app,” and “walking habits” (Table 2). The qualitative data suggested that although this was useful for reading initial information about the benefits of brisk walking and particularly for downloading the app, some participants reported limited recollection of the leaflet at the follow-up point:

I did have a quick flick through it. And obviously the bit about finding the app.

Female participant; aged 49 y

T1 questionnaire results indicated a mean usefulness of 4.1 (SD 1) for the app, but with higher ratings among the 33 participants still using it compared to the 5 who had ceased. Most (28/38, 74%) of the users found it extremely useful or very useful. Participants who reported still using the app reported higher perceived accuracy of the app in recording their time spent walking compared to those not using the app anymore (Table 2). This is supported by the qualitative findings, with participants reporting still using the app and finding it enjoyable to monitor their progress on it:

I use it all the time now...I still want to make sure I have got at least two cups.

Male participant; aged 74 y

The usefulness of the planner explored in the qualitative interviews highlighted that although the planners were useful to get started and to form habits, their use ceased over time due to factors such as not finding planning as helpful long-term, finding the app sufficient to motivate them, and just forgetting to use them:

I think you need the planner thing for the first week or so but after that I don’t think you do.

Female participant; aged 56 y

###### Perceived Benefits of Engagement With the Intervention

Where participants felt they were benefiting from taking part, they reported feeling motivated to continue their brisk walking, reporting feelings of enjoyment, better mood and well-being, and improvements in physical health and fitness:

I definitely felt better in myself, there’s no question about that. I felt fitter as well.

Male participant; aged 60 y

As well as the direct impact of the intervention, many participants also reported feeling more able to engage in activities of daily living, such as going shopping, socializing, and doing housework because of their improved fitness and well-being:

I have started going out with more friends...getting out a bit more and feel better and everything.

Female participant; aged 67 y

## Discussion

### Summary of Findings

This study combined data collected as part of the APPROACH pilot RCT to assess the implementation of a multicomponent, app-based behavioral intervention to promote brisk walking in people LWBC. The findings of this process evaluation demonstrated proficient implementation of the intervention and suggest that there are several mechanisms of impact underlying the efficacy of the intervention as well as contextual factors that can be barriers or facilitators to engagement.

### Successful Implementation of the APPROACH Intervention

The successful delivery of intervention components and intended BCTs is essential for attributing any changes in behavior to the intervention in question [41]. This study demonstrated proficient implementation, with most participants reporting engagement with the intervention components, including downloading the app, reading the leaflet, receiving the behavioral support call, and using the planners. In addition, fidelity is rarely reported in evaluations of PA interventions [29]. In a systematic review of 21 studies assessing the quality of measuring delivery fidelity in PA interventions, Lambert et al [42] reported considerable heterogeneity in evaluating delivery fidelity. This study reports high delivery fidelity of the intended BCTs in the behavioral support calls [21]. While interventions with only a single intervention facilitator often exhibit higher fidelity, investigating how various intervention facilitators across different contextual settings deliver BCTs is essential for understanding the real-world application and scalability of interventions [43]. Particularly where multiple intervention facilitators are involved in the delivery, future research should consider factors such as personal characteristics and individual context when evaluating intervention delivery [44,45].

The BCTs called information on health consequences, action planning, and habit formation showed the highest fidelity, and these BCTs appear to influence key mechanisms of impact highlighted by participants in the qualitative interviews. Empowering participants with information on why they should go walking and providing them with information about its benefits appeared to enhance their engagement and adherence to their planned walks. The delivery of and engagement with the action planning BCT is promising, as this BCT was shown to be associated with larger effect sizes in a systematic review of 26 studies evaluating BCT effectiveness in PA interventions in adults who are healthy and inactive [29]. This study extends this finding and suggests that this BCT is suitable and appropriate for people LWBC and should be included in future intervention designs with this population. The BCTs that were not successfully delivered included those called nonspecific reward, nonspecific incentive, and framing or reframing. Despite the BCT called nonspecific reward receiving a low fidelity rating in the recorded behavioral support calls, the qualitative interviews suggested that this BCT was effectively covered in other aspects of the intervention, with participants reporting enjoying working toward the rewards and trophies in the NHS Active 10 app. The low delivery fidelity of the BCT called framing/reframing could be attributed to participants’ voluntary enrollment in a PA trial. It is likely that they already recognized the importance of PA even without fully recognizing its role in life beyond a cancer diagnosis [46,47].

### BCTs Underlying Change

The mechanisms of impact identified by participants in the qualitative interviews and follow-up questionnaire were in line with previous research. For instance, self-monitoring of behavior is one of the most frequently used components in complex PA interventions [48], and multiple systematic reviews have demonstrated its effectiveness in increasing PA that is maintained long-term [49-51]. In this intervention, the NHS Active 10 app allowed participants to track their brisk walking and total walking, and participants reported that being able to see their improvement over time motivated them to continue their behaviors. In a similar pilot RCT using a self-monitoring app alone, Ormel et al [52] reported that participants in the intervention group increased their activity more from baseline to 6 weeks, but this difference was not maintained at 12 weeks. The authors attributed this to a potential loss of novelty and interest in the app. Although not powered to detect differences in PA, the findings of this study suggest that the second behavioral support call was important in continuing encouragement of monitoring of behavior and reviewing of goals, with participants describing a sense of accountability with the later call. This feedback indicates that a light-touch intervention call to check in with participants can be beneficial in consolidating commitment to PA goals in people LWBC. While technology can help reduce resource demands, future research should consider the benefits of a low-burden behavioral support call to augment an app as an already powerful and scalable intervention component.

Participants extensively discussed the app as the driving component of the intervention, supporting our previous reports of high engagement with the NHS Active 10 app [22]. The discussions revealed that another mechanism underlying behavior change in this context was the ability of the app to increase motivation toward reward using gamification techniques [53]. The performance of the desired behavior (brisk walking) was reinforced by the positive feelings of encouragement and dedication resulting from these cups and trophies in the app.

### Encouraging Habit Formation

The APPROACH intervention was informed by habit theory [21], and the identification of habit formation as a mechanism of impact in both the qualitative interviews and questionnaire data demonstrates that this BCT was delivered effectively in the intervention. Participants reported that the leaflet, app, behavioral support call, and planners helped to establish sustainable walking habits. Gardner et al [54] define habit formation as “learning cue–behaviour associations, that when cued, automatically generate action impulses.” This sense of automaticity was described by participants in the qualitative interviews, whereby consistent repetition of their walking daily led to the enactment of brisk walking. This meant that they engaged in brisk walking even during activities that previously would not have involved this exercise intensity. These findings are also supported by the increased SRBAI results from baseline to follow-up, which showed an increase in the initiation of walks as well as the way walking was executed (ie, briskly) [55]. The importance of encouraging long-term engagement in PA after the intervention period is highlighted in systematic reviews of PA maintenance in cancer populations that report only modest improvements at longer follow-up time points [30,56]. The results of this study endorse the integration of habit theory into future interventions aimed at increasing PA to overcome the challenge of sustaining behavioral changes over time [56].

To reinforce the idea of habit, the walking planner cards were designed to promote habit formation, facilitate planning, and prompt participants to engage in PA [21,54]. However, both questionnaire data and interview data showed that the structured design of the planner cards was not compatible with the day-to-day changing schedules and lifestyles of some participants, highlighting the importance of conducting this process evaluation to account for and reconsider this contextual aspect of the intervention and future similar interventions. The need for flexibility in lifestyle and planning was reported as a barrier by participants and further confirmed by the reflections of the intervention facilitator (CB) after discussing their use with participants.

### Barriers to Engagement: Cancer Impact and Competing Commitments

Other contextual barriers included the impact of cancer and its treatment, having other competing commitments, technical difficulties with the app, and environmental influences. The side effects of cancer and its treatment have previously been identified as a key barrier to PA participation in systematic reviews [18] as well as in our own preparatory work for this pilot RCT [19]. Participants in this study reported that the impact of cancer and its treatment inhibited their ability to engage in some elements of the intervention due to different physiological, structural, and psychological factors. Cancer-related fatigue is the most reported symptom in people LWBC who have undergone treatment with prevalence estimates of up to 90% of those treated with radiotherapy and 80% of those treated with chemotherapy [57,58]. In this study, participants discussed fatigue symptoms and felt that engaging with the intervention during treatment would have been difficult. Beyond this physiological barrier, the structural barrier of having any appointments for their cancer care also reduced their ability to engage with some components, including the planner card, as there were many hospital appointments that they had to attend and plan around. We have previously reported on the perceived suitability of the timing of the APPROACH intervention, with most participants feeling that it was reasonable [22]. While some participants felt that engaging during treatment would be difficult, others felt that it was useful to have something else to focus on and have control over [22]. Previous reviews in this area have also reported discrepancies in the preferred timing of PA intervention delivery within the cancer care pathway [18,59]. Involvement in PA at an earlier stage has been associated with improved treatment response, tolerance, and quality of life [13,60,61]. Considering, the delivery of the APPROACH intervention during and after the treatment for cancer is still endorsed while recognizing the contextual barriers, such as this, when interpreting APPROACH intervention outcomes. These findings highlight the importance of involving patient perspectives in future intervention design with this population, with the acknowledgment that different stages of the treatment pathway can facilitate or inhibit PA participation and should be accounted for when assessing intervention delivery and engagement.

### Driving Engagement: Support, Trust, and Perceived Benefits

Facilitators to engagement included having support from others, trust in health care professionals, the perceived usefulness of the intervention, and the perceived benefits of engagement with the intervention. The BCTs called social support (practical) and social support (emotional) were competently delivered in line with the protocol [21,31]. Accordingly, participants recognized how having support from others, such as family members and partners, enhanced their engagement with the intervention and how they felt supported by the intervention facilitator. Social support has previously been identified as highly important in PA engagement [62], with reasons, such as accountability, being cited as helping to facilitate and promote engagement [63]. In our preparatory work for this RCT, trust in health care professionals emerged as a crucial factor influencing engagement [19], and this was highlighted again in this study, where incorporating an endorsement letter from the clinical care team enhanced app credibility.

### Limitations

Limitations of this study include that only 1 intervention facilitator delivered all behavioral support calls, which may explain the high-fidelity ratings. It is crucial to consider the transferability of the intervention across individuals, particularly when envisioning integration into routine NHS care [20,22]. In addition, there may be some recall bias influencing results, as most of the questionnaire data were collected at the 3-month follow-up [64]. Participants may have had difficulty in answering questions on earlier components (eg, leaflet information) compared to components they were still using (eg, app). Due to the pilot nature of this study, where feasibility and acceptability were the main outcomes of interest, we were unable to examine how engagement with each intervention component influenced the primary outcome of brisk walking. For future research, the application of the Multiphase Optimization Strategy with a factorial design could offer more insights into how differing engagement with each component can impact the main outcomes and help inform intervention optimization for larger efficacy RCTs [65,66]. Finally, despite its recognition as a potential mechanism, it is difficult to assess how the intervention helps to establish longer-term habits, which are key for PA maintenance [67], as this pilot RCT only examined outcomes at 3 months.

### Conclusions

This study extends our previously published findings on the APPROACH pilot RCT [22] by demonstrating that the intervention was delivered as intended with high levels of engagement from participants. In addition, this paper highlighted the potential mechanisms through which change occurs, such as habit formation and behavioral monitoring, which are in line with the intended BCTs used in this intervention. The process evaluation also highlighted important contextual factors to consider when progressing to the APPROACH main trial, including facilitators, such as social support, which played a significant role in promoting adherence to the intervention. The protocol for the definitive RCT will report on adaptations made to APPROACH based on the feedback gathered in this study. This process evaluation provides strong support for the progression to the stage-3, definitive RCT to evaluate the effectiveness of the APPROACH intervention (began in November 2023) and enables a more nuanced understanding of how the APPROACH intervention works and the contextual factors to consider with implementation.

## Appendix

Multimedia Appendix 1 APPROACH intervention call behavior change technique checklist, intended delivery technique, and mean delivery fidelity score.

Multimedia Appendix 2 Adapted Dreyfus rating scale for the intervention behavior change technique calls.

Multimedia Appendix 3 Self-Report Behavioural Automaticity Index.

Multimedia Appendix 4 Exit interview guide.

## Abbreviations

BCT: behavior change technique
CONSORT: Consolidated Standards of Reporting Trials
LWBC: living with and beyond cancer
MVPA: moderate-to-vigorous physical activity
PA: physical activity
RCT: randomized controlled trial
SRBAI: Self-Report Behavioural Automaticity Index
T0: time point 0
T1: time point 1
