# Supplementary material for: An App-Based Intervention With Behavioral Support to Promote Brisk Walking in People Diagnosed With Breast, Prostate, or Colorectal Cancer (APPROACH): Process Evaluation Study
Source: JMIR Cancer. 2025 Feb 10;11:e64747. doi: 10.2196/64747 (PMC11851027; doi:10.2196/64747)
Supplement: Multimedia Appendix 1 [file cancer_v11i1e64747_app1.docx]

| APPROACH intervention call behavior change technique checklist, intended delivery technique and mean delivery fidelity score (N=42^a^) | | |
| --- | --- | --- |
| Behavior Change Techniques | Intended technique delivery | Mean Delivery Fidelity Score (SD)^b^ |
|  |  |  |
| 1. Credible source | Introduce self as working in a clinical team/ state that information has come from government guidelines | 4.86 (0.52) |
| 1. Information about health consequences | Discuss physical benefits of PA | 4.98 (0.15) |
| 1. Information about emotional consequences | Discuss mental health benefits of PA | 4.69 (0.78) |
| 1. Pros and Cons | Discuss motivations and concerns about increasing brisk walking | 4.48 (0.67) |
| 1. Problem solving | Help to overcome any concerns about brisk walking | 4.21 (0.87) |
| 1. Information about others approval | Tell them why we are recommending, other cancer patients have recommended. | 4.40 (0.83) |
| 1. Instruction on how to form a behavior | Describe brisk walking | 4.60 (0.89) |
| 1. Behavioral experiments | Suggest trying it to see if it increases confidence | 4.14 (0.81) |
| 1. Verbal persuasion about capability | Tell them it is possible for them to do this, and others have done it to | 3.90 (0.93) |
| 1. Behavioral practice/ rehearsal | Promote habit formation for initiating a walk | 4.81 (0.45) |
| 1. Habit formation | Explain how habit formation works and how it will help them in this study | 4.88 (0.40) |
| 1. Action planning | Make an action plan | 4.90 (0.30) |
| 1. Self-incentive | Promote self-reward during and/ or after walking | 4.43 (1.11) |
| 1. Self-reward | Promote self-reward during and/ or after walking | 4.18 (1.17) |
| 1. Non-specific reward | Promote non-specific reward self-reward during and/ or after walking | 2.71 (1.42) |
| 1. Non-specific incentive | Promote non-specific reward self-reward during and/ or after walking | 2.69 (1.37) |
| 1. Feedback on behavior | Promote using the app to track activity | 4.71 (0.94) |
| 1. Prompt/ cues | Promote setting prompts to remind and encourage them to walk | 3.69 (1.77) |
| 1. Goal setting | Set a number of active 10’s | 4.76 (0.85) |
| 1. Social support (practical) | Promote asking friends to walk with them | 3.79 (1.42) |
| 1. Social support (emotional) | Promote asking friends to support them | 3.57 (1.43) |
| 1. Self-monitoring | Promote using the walking planner | 4.71 (0.89) |
| 1. Framing/ reframing | Prompt participants to use the information provided to overcome their concerns about exercise | 1.31 (1.47) |
| 1. Review behaviour goals | Remind them of their target | 4.60 (1.31) |
| 1. Discrepancy between current behavior and goal | Ask how they are getting on with their target | 4.55 (1.31) |
| ^a^One participant did not receive either call; One participant was excluded due to a recording error on call 1 so neither of their calls were included in fidelity results.  ^b^Possible rating range was 0-5. | | |
