# Supplementary material for: An App-Based Intervention With Behavioral Support to Promote Brisk Walking in People Diagnosed With Breast, Prostate, or Colorectal Cancer (APPROACH): Process Evaluation Study
Source: JMIR Cancer. 2025 Feb 10;11:e64747. doi: 10.2196/64747 (PMC11851027; doi:10.2196/64747)
Supplement: Multimedia Appendix 2 [file cancer_v11i1e64747_app2.docx]

| Adapted Dreyfus rating scale for the intervention behavior change technique calls (1) | | |
| --- | --- | --- |
| Scoring | Examples | Delivery Fidelity Category |
| 0 | Absence of feature and/ or highly inappropriate performance | Low fidelity |
| 1 | Minimal use of feature and/ or inappropriate performance | Low fidelity |
| 2 | Scope for improvement, alongside numerous minor and some major inconsistencies | Scope for improvement |
| 3 | Competent, good features but some minor inconsistencies or problems | Competent |
| 4 | Very good features, few inconsistencies or problems | Proficient |
| 5 | Excellent features, no problems or inconsistencies | Expert |

**References**

1. Cross R, Greaves CJ, Withall J, Rejeski WJ, Stathi A. Delivery fidelity of the REACT (REtirement in ACTion) physical activity and behaviour maintenance intervention for community dwelling older people with mobility limitations. BMC Public Health. 2022;22(1):1112.
