# Supplementary material for: An App-Based Intervention With Behavioral Support to Promote Brisk Walking in People Diagnosed With Breast, Prostate, or Colorectal Cancer (APPROACH): Process Evaluation Study
Source: JMIR Cancer. 2025 Feb 10;11:e64747. doi: 10.2196/64747 (PMC11851027; doi:10.2196/64747)
Supplement: Multimedia Appendix 3 [file cancer_v11i1e64747_app3.docx]

#### Self-Report Behavioural Automaticity Index (SRBAI) (1)

**Going for a walk is something…**

1. **I do automatically**

| Disagree |  | Neither agree nor disagree | | |  | Agree |
| --- | --- | --- | --- | --- | --- | --- |
| 1 | 2 | 3 | 4 | 5 | 6 | 7 |
| ❑ | ❑ | ❑ | ❑ | ❑ | ❑ | ❑ |

1. **I do without having to consciously remember**

| Disagree |  | Neither agree nor disagree | | |  | Agree |
| --- | --- | --- | --- | --- | --- | --- |
| 1 | 2 | 3 | 4 | 5 | 6 | 7 |
| ❑ | ❑ | ❑ | ❑ | ❑ | ❑ | ❑ |

1. **I do without thinking**

| Disagree |  | Neither agree nor disagree | | |  | Agree |
| --- | --- | --- | --- | --- | --- | --- |
| 1 | 2 | 3 | 4 | 5 | 6 | 7 |
| ❑ | ❑ | ❑ | ❑ | ❑ | ❑ | ❑ |

1. **I start doing before I realise I’m doing it**

| Disagree |  | Neither agree nor disagree | | |  | Agree |
| --- | --- | --- | --- | --- | --- | --- |
| 1 | 2 | 3 | 4 | 5 | 6 | 7 |
| ❑ | ❑ | ❑ | ❑ | ❑ | ❑ | ❑ |

**Walking briskly (you breathe a bit faster and feel a bit warmer) is something…**

1. **I do automatically**

| Disagree |  | Neither agree nor disagree | | |  | Agree |
| --- | --- | --- | --- | --- | --- | --- |
| 1 | 2 | 3 | 4 | 5 | 6 | 7 |
| ❑ | ❑ | ❑ | ❑ | ❑ | ❑ | ❑ |

1. **I do without having to consciously remember**

| Disagree |  | Neither agree nor disagree | | |  | Agree |
| --- | --- | --- | --- | --- | --- | --- |
| 1 | 2 | 3 | 4 | 5 | 6 | 7 |
| ❑ | ❑ | ❑ | ❑ | ❑ | ❑ | ❑ |

1. **I do without thinking**

| Disagree |  | Neither agree nor disagree | | |  | Agree |
| --- | --- | --- | --- | --- | --- | --- |
| 1 | 2 | 3 | 4 | 5 | 6 | 7 |
| ❑ | ❑ | ❑ | ❑ | ❑ | ❑ | ❑ |

1. **I start doing before I realise I’m doing it**

| Disagree |  | Neither agree nor disagree | | |  | Agree |
| --- | --- | --- | --- | --- | --- | --- |
| 1 | 2 | 3 | 4 | 5 | 6 | 7 |
| ❑ | ❑ | ❑ | ❑ | ❑ | ❑ | ❑ |

**References**

1. Gardner B, Abraham C, Lally P, de Bruijn G-J. Towards parsimony in habit measurement: Testing the convergent and predictive validity of an automaticity subscale of the Self-Report Habit Index. International Journal of Behavioral Nutrition and Physical Activity. 2012;9(1):102.
